# Supplementary material for: Single‐Molecule FRET‐Tracking of InlB‐Activated MET Receptors in Living Cells
Source: Small. 2025 Dec 19;22(5):e07115. doi: 10.1002/smll.202507115 (PMC12824565; doi:10.1002/smll.202507115)
Supplement: Supplementary file 1 — Supporting Information [file SMLL-22-e07115-s003.pdf]

NANO · MICRO
small

Supporting Information

for *Small*, DOI 10.1002/smll.202507115

Single-Molecule FRET-Tracking of InlB-Activated MET Receptors in Living Cells

*Yunqing Li, Marina S. Dietz, Hans-Dieter Barth, Hartmut H. Niemann and Mike Heilemann**

Supplementary Material

Single-Molecule FRET-Tracking of InlB-Activated MET Receptors in Living Cells

Yunqing Li[1], Marina S. Dietz[1], Hans-Dieter Barth[1], Hartmut H. Niemann[2], Mike Heilemann[1,*]

[1]Institute of Physical and Theoretical Chemistry, Goethe University Frankfurt, 60438, Frankfurt am Main, Germany
[2]Department of Chemistry, Bielefeld University, Bielefeld, 33615, Germany

*Correspondence: heilemann@chemie.uni-frankfurt.de

Supplementary Movies

Supplementary Movie 1: Single-molecule FRET movie of MET receptors labeled with InlB-T-Cy3B and InlB-T-ATTO 647N. Scale bar 1 µm. Corresponding to Figure S1A, upper panel.

Supplementary Movie 2: Single-molecule FRET movie of MET receptors labeled with InlB-T-Cy3B and InlB-T-ATTO 647N. Scale bar 1 µm. Corresponding to Figure S1A, bottom panel.

Supplementary Movie 3: Single-molecule FRET movie of MET receptors labeled with InlB-H-Cy3B and InlB-T-ATTO 647N. Scale bar 1 µm. Corresponding to Figure S1B, upper panel.

Supplementary Movie 4: Single-molecule FRET movie of MET receptors labeled with InlB-H-Cy3B and InlB-T-ATTO 647N. Scale bar 1 µm. Corresponding to Figure S1B, bottom panel.

Supplementary Figures

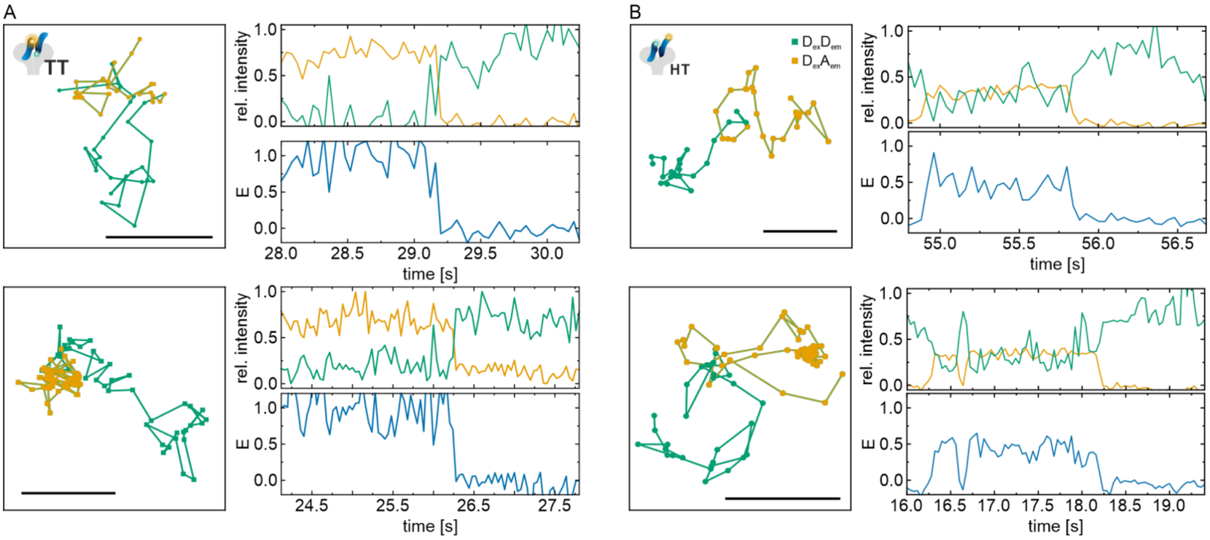

Figure S1: smFRET-RAP of (MET:InlB$_{321}$)$_2$ dimers in living U-2 OS cells. Exemplary smFRET trajectories for **A**) Cy3B-T-InlB$_{321}$ and ATTO 647N-T-InlB$_{321}$ and **B**) Cy3B-H-InlB$_{321}$ and ATTO 647N-T-InlB$_{321}$ (right). Trajectories are shown with the donor (green) and acceptor (orange) intensity traces upon donor excitation and the respective FRET efficiencies (blue). Scale bars are 500 nm.

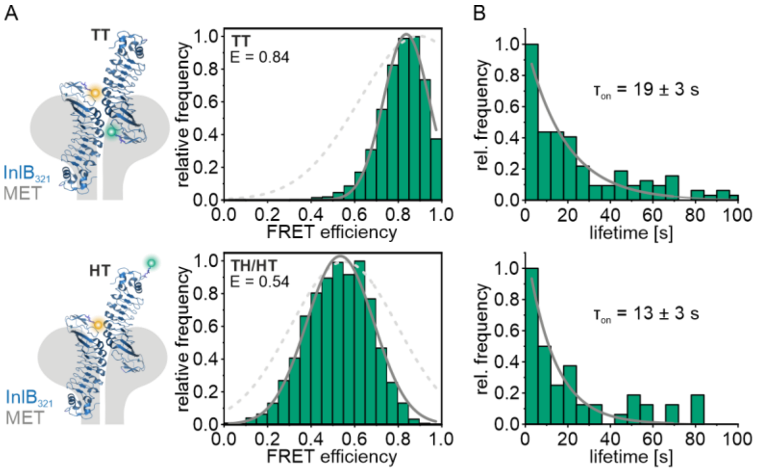

Figure S2: smFRET of (MET:InlB$_{321}$)$_2$ dimers in fixed cells. A) FRET efficiency distributions for Cy3B-T-InlB$_{321}$/ATTO 647N-T-InlB$_{321}$ (N = 113 smFRET traces from 64 cells) and Cy3B-H-InlB$_{321}$/ATTO 647N-T-InlB$_{321}$ (N = 49 smFRET traces from 39 cells) are displayed. Overlay is the FRET efficiency distribution of living cells (light gray dotted line). **B**) The corresponding lifetimes of smFRET traces were histogrammed and fitted with a single-exponential decay.

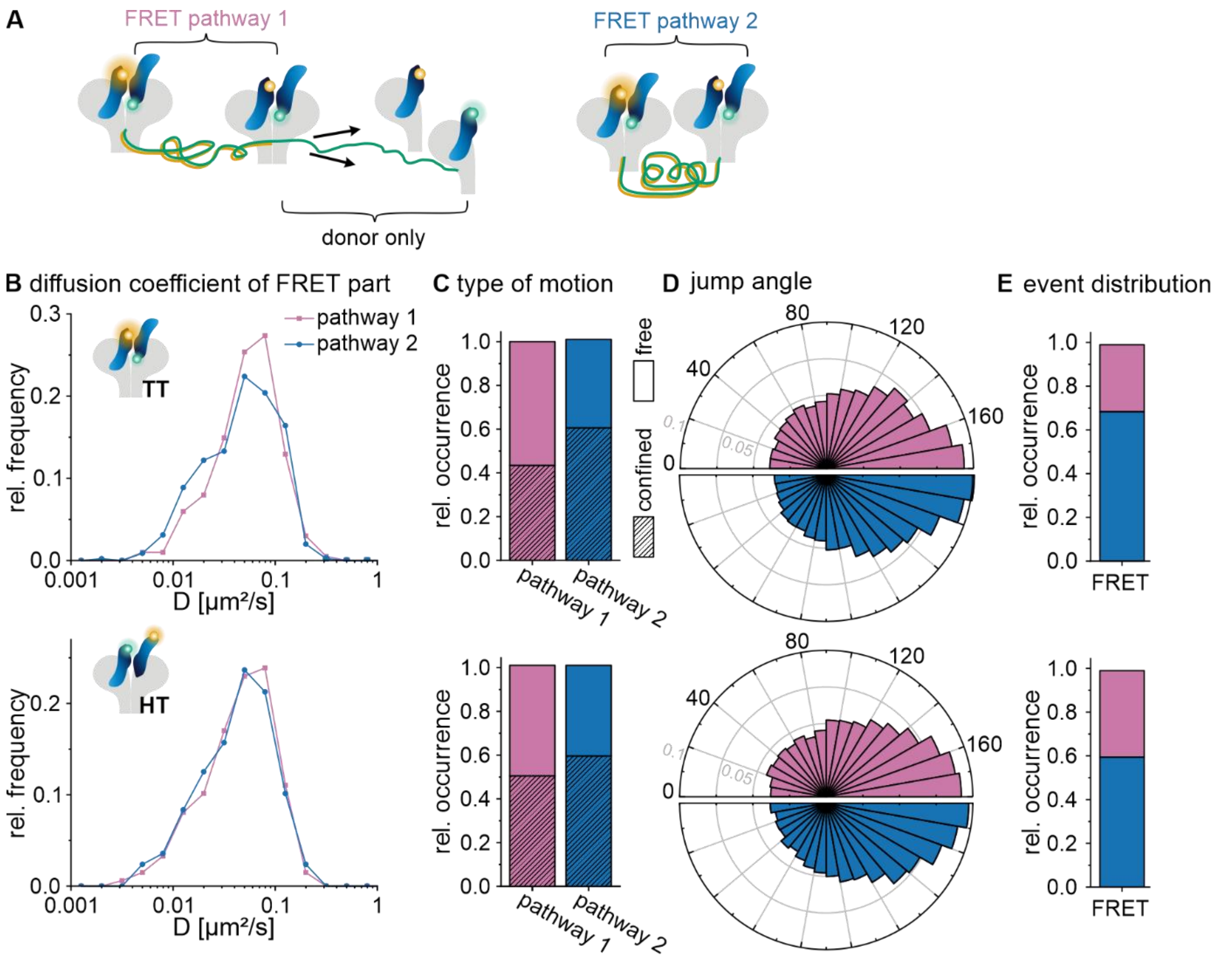

Figure S3: Dynamics of (MET:InlB$_{321}$)$_2$ dimer detected with smFRET in two pathways. A) Schematic of two possible pathways observed in single-molecule FRET trajectories. **B**) Distribution of diffusion coefficients determined for the FRET segments in single-molecule trajectories for pathway 1 (pink) and pathway 2 (blue) (N = 451 trajectories from 27 cells (InlB T-T) and 503 trajectories from 24 cells (InlB H-T), respectively, and from at least 3 independent experiments). **C**) Relative occurrence of restricted and free diffusion in FRET segments of pathway 1 and 2. **D**) Distribution of jump angles of FRET segments of single-molecule trajectories in pathways 1 and 2 (grey scales represent relative frequencies). **E**) Relative occurrence of pathway 1 and pathway 2 in single-molecule trajectories.

Supplementary Table

Table S1: Degree of labeling of fluorophore-labeled InlB$_{321}$ variants. InlB variants were labeled either with Cy3B or ATTO 647N maleimide. The cysteine mutations used for fluorophore labeling are indicated. The degree of labeling (DOL) was determined by absorption spectroscopy.

Variant	Mutation	DOL (%)
InlB-T-Cy3B	K280C	87
InlB-T-ATTO 647N	K280C	69
InlB-H-Cy3B	K64C	70
InlB-H-ATTO 647N	K64C	103

Supplementary Note

Supplementary Note 1. MET dimers can either reside on the plasma membrane or inside the cytosol in endocytic pits or clathrin-coated pits (CCPs). In this study, we reason that the MET dimers we observe are *bona fide* located in the plasma membrane.

First, for endosome-located MET receptors, we expect that single-particle trajectories would quickly disappear from the TIRF observation window and thus be very short. The depth of the evanescent field in our TIRF settings is around 100 nm; however, since the basal plasma membrane is slightly elevated above the glass surface, the actual observable volume inside a cell is likely even smaller.[1] We excluded such intracellular trajectories from our analysis by applying a minimum trajectory length threshold of 20 frames (0.8 seconds). In the case of 3D diffusion, the probability of intracellular movement occurring for 20 steps within the tiny z range is negligibly small. Second, CCPs assemble directly at the plasma membrane before undergoing endocytosis. These forming CCPs are predominantly immobile. We can therefore also exclude the localization of MET receptor dimers in CCPs, given the negligible immobile fraction (<1%) of the detected trajectories and the absence of particles with diffusion coefficients below 0.001 $\mu m^2/s$. In contrast, CCPs typically exhibit diffusion coefficients around 0.001 $\mu m^2/s$,[2] and proteins associated with CCPs often display diffusion coefficients close to or even below this value.[3-5]

References

1. Asher WB, Geggier P, Holsey MD, Gilmore GT, Pati AK, Meszaros J, et al. Single-molecule FRET imaging of GPCR dimers in living cells. Nat Methods. 2021 Apr;18(4):397–405.
2. Liu AP, Loerke D, Schmid SL, Danuser G. Global and local regulation of clathrin-coated pit dynamics detected on patterned substrates. Biophys J. 2009 Aug 19;97(4):1038–47.
3. Eichel K, Jullié D, Barsi-Rhyne B, Latorraca NR, Masureel M, Sibarita JB, et al. Catalytic activation of β-arrestin by GPCRs. Nature. 2018 May;557(7705):381–6.
4. Ibach J, Radon Y, Gelléri M, Sonntag MH, Brunsveld L, Bastiaens PIH, et al. Single Particle Tracking Reveals that EGFR Signaling Activity Is Amplified in Clathrin-Coated Pits. PLoS One. 2015 Nov 17;10(11):e0143162.
5. Yanagawa M, Hiroshima M, Togashi Y, Abe M, Yamashita T, Shichida Y, et al. Single-molecule diffusion-based estimation of ligand effects on G protein-coupled receptors. Sci Signal [Internet]. 2018 Sep 18;11(548). Available from: http://dx.doi.org/10.1126/scisignal.aao1917
